# An Integrative Co-localization (INCO) Analysis for SNV and CNV Genomic Features With an Application to Taiwan Biobank Data

**DOI:** 10.3389/fgene.2021.709555

**Published:** 2021-09-08

**Authors:** Qi-You Yu, Tzu-Pin Lu, Tzu-Hung Hsiao, Ching-Heng Lin, Chi-Yun Wu, Jung-Ying Tzeng, Chuhsing Kate Hsiao

**Affiliations:** ^1^Institute of Epidemiology and Preventive Medicine, College of Public Health, National Taiwan University, Taipei, Taiwan; ^2^Department of Public Health, National Taiwan University, Taipei, Taiwan; ^3^Department of Medical Research, Taichung Veterans General Hospital, Taichung, Taiwan; ^4^Graduate Group in Genomics and Computational Biology, University of Pennsylvania, Philadelphia, PA, United States; ^5^Department of Statistics, University of Pennsylvania, Philadelphia, PA, United States; ^6^Department of Statistics and Bioinformatics Research Center, North Carolina State University, Raleigh, NC, United States

**Keywords:** co-localization, gene-level, integrative analysis, Taiwan Biobank, CNV, SNV, CNV-SNV cross-platform interaction, rare variant

## Abstract

Genomic studies have been a major approach to elucidating disease etiology and to exploring potential targets for treatments of many complex diseases. Statistical analyses in these studies often face the challenges of multiplicity, weak signals, and the nature of dependence among genetic markers. This situation becomes even more complicated when multi-omics data are available. To integrate the data from different platforms, various integrative analyses have been adopted, ranging from the direct union or intersection operation on sets derived from different single-platform analysis to complex hierarchical multi-level models. The former ignores the biological relationship between molecules while the latter can be hard to interpret. We propose in this study an integrative approach that combines both single nucleotide variants (SNVs) and copy number variations (CNVs) in the same genomic unit to co-localize the concurrent effect and to deal with the sparsity due to rare variants. This approach is illustrated with simulation studies to evaluate its performance and is applied to low-density lipoprotein cholesterol and triglyceride measurements from Taiwan Biobank. The results show that the proposed method can more effectively detect the collective effect from both SNVs and CNVs compared to traditional methods. For the biobank analysis, the identified genetic regions including the gene *VNN2* could be novel and deserve further investigation.

## Introduction

Many genomic studies based on DNA single nucleotide variants (SNVs) have been conducted to explore mechanisms of disease etiology, evaluate treatment effects, and identify potential drivers of disease. For instance, through genome-wide association studies (GWASs), several trait-associated single nucleotide polymorphisms (SNPs) have been detected in past decades (Wei et al., 2010; Network et al., 2015; Juang et al., 2019). However, issues arise in traditional GWAS analyses. For example, the existence of linkage disequilibrium (LD) may lead to the discovery of non-causal DNA markers. In addition, when signals are weak, current statistical models may not have enough power to detect biomarkers, especially when the signal comes from a rare variant and/or when sample size is limited. Furthermore, GWAS findings may only partially account for the heritability of complex diseases, leaving a large proportion of heritability unexplained.

To avoid or reduce concerns related to the above issues, several approaches have been adopted. Examples include, but are not limited to, the incorporation of multi-omics data (Sun and Hu, 2016; Hasin and Lusis, 2017; Eddy et al., 2020), the development of statistical methodology for uncovering missing heritability (Zuk et al., 2012; Yang et al., 2015; Young, 2019), and the examination of gene-gene and gene-environment interactions (van IJzendoorn et al., 2011; Kaprio, 2012; Zhou et al., 2021). For omics data from multiple platforms, the current advancement in high-throughput genetic technology has facilitated the collection of genomic information from more than one single platform, such as DNA SNVs and copy number variations (CNVs) from SNP microarrays and/or next-generation sequencing. It is expected that, through the combination of different types of omics data, scientists can better capture or dissect the genetic contribution in a specific disease. Some successful applications include several breast cancer studies (Beckmann et al., 2007; Curtis et al., 2012; Zahnleiter et al., 2013; Hendrickx et al., 2017), a prostate cancer study (Taylor et al., 2010), and a study of mental disorders (Wang et al., 2019). Review articles about such integrated analysis have received great attention recently (see for instance Richardson et al., 2016; Sun and Hu, 2016; and Wu et al., 2019, among many others and references therein). Some have focused on the biological perspective (Hasin and Lusis, 2017; Eddy et al., 2020), some on statistical methodology in general (Richardson et al., 2016; Sun and Hu, 2016), and some on the perspective of variable selection and clustering (Wu et al., 2019).

Among possible omics data to be concurrently analyzed with DNA SNVs, CNV is a common choice, since it has been observed across all human genomes and found causative in several phenotypic traits, by influencing the downstream gene expression levels (Curtis et al., 2012; Li et al., 2013; Zahnleiter et al., 2013). In gene expression profiles, it is estimated around 17% of the variation can be explained by the corresponding CNVs (Stranger et al., 2007). However, similar to the issues in GWASs, it is difficult to identify directly a CNV of large impact for one specific disease, since CNV has the properties of low prevalence and multi-scale features. One solution is to analyze multiple CNVs simultaneously. Algorithms based on multiple CNVs have been proposed, such as the CNV kernel association test (CKAT) (Zhan et al., 2016), the CNV Collapsing Random Effects Test (CCRET) (Tzeng et al., 2015) and the copy number profile curve-based association test (CONCUR) (Brucker et al., 2020). Successful examples using multiple CNVs have been reported in obesity and psychiatric disorders (Lee K.W. et al., 2012; Carpenter et al., 2015).

Both CNVs and SNVs play important roles in complex diseases such as cancer. An association study with both SNVs and CNVs included as biomarkers may increase the statistical power. Such study designs have produced important findings. For example, Beckmann et al. (2007) found mutant alleles in *BRCA1* and *BRCA2*. It was reported that copy number variation exists in early-onset breast cancer patients (Krepischi et al., 2012; Pan et al., 2019). Another breast cancer study reported a possible explanation of gene expression changes by somatic CNVs and several putative cancer genes with copy number deletion (Curtis et al., 2012). Intriguingly, many DNA sequence mutants, especially the loss-of-function alleles, can be identified in the vicinity of CNVs (Hastings et al., 2009), and CNVs frequently cluster together instead of randomly distributing across the genome. These findings suggest that an integrated analysis with both SNVs and CNVs together may better capture their collective influence on the outcome variable (Liu et al., 2018). Approaches in this direction include studies that sequentially analyze SNVs and CNVs, such as the breast cancer research project and studies of human height (Beckmann et al., 2007; Curtis et al., 2012; Zahnleiter et al., 2013). This integrative approach may be crucial in refining a region for further investigation of causal variants.

Traditional integrated analyses of multi-omics data often analyze each single platform separately to identify a set of statistically significant signals, and then take the naïve union or intersection of these resulting sets as the final finding for potential candidate genes (Myocardial Infarction Genetics Consortium et al., 2009; Curtis et al., 2012; Li et al., 2013). This analysis scheme considers each omics data platform in a parallel sense (Wu et al., 2019). Such an approach is based on set operations and may fail to identify causal variants, especially when the trait is driven concurrently by both SNVs and CNVs and not marginally associated with either of the two types of biomarkers. As an alternative, methods that examine simultaneously both types of markers have been adopted. Such a scheme is termed as hierarchical or vertical (Richardson et al., 2016; Wang et al., 2019; Wu et al., 2019). For instance, Gamazon et al. (2014) suggested the use of a single SNV association test with the copy number status as a covariate to achieve better performance. Different from the single gene and/or single-variant approach, Momtaz et al. (2018) proposed analyzing the multiple variants at the gene-set or pathway level simultaneously. They all noted that a joint analysis of SNVs and CNVs would be a favorable approach in exploring genetic contributions to complex diseases. Most current integration studies analyze omics data with a vertical scheme. In a vertical integration study, different genomic profiling from the same subject is collected; while in a horizontal integration study, such as the meta-analysis of multiple GWAS datasets, the collection of the same type of omics data from different subjects in multiple studies is collected (Richardson et al., 2016; Sun and Hu, 2016; Wang et al., 2019; Wu et al., 2019).

The integration analysis we consider here involves omics data of different molecular levels collected from the same individual. We propose in this study an efficient integrative co-localization (INCO) algorithm to integrate SNVs and CNVs and to provide a refined genetic region for identification of causal variants. The proposed INCO is a hybrid approach and has several advantages. First, the INCO starts with a screening procedure at the gene-level to preserve the biological interpretation while reducing the dimensionality. Second, the INCO incorporates the effects of SNVs and CNVs in a collective way rather than in a parallel manner, so that the concurrent effect from both types of markers can be modeled. This differs from the interaction studies where the main effects of both SNVs and CNVs have to be significant before modeling the interaction effect. Third, though the INCO starts the analysis at the gene-level, its final procedure focuses on narrower genetic regions. Such an arrangement can alleviate the burden of multiplicity on statistical power and guard against the sparsity problem due to rare variants. The methodology of INCO will be explained in Section 2, followed by simulation studies to evaluate its performance and to compare it with other existing methods. Then the INCO method will be applied to the Taiwan Biobank data (TWB), focusing on low-density lipoprotein cholesterol (LDL-C) and triglyceride (TG) levels.

## Method

### Notation and the INCO Algorithm

Let *K* denote the type of genomic variants in the target genomic region. For instance, *K* = 1 refers to SNVs and *K* = 2 to CNVs. For any fixed gene, the genetic information of the *i*-th (*i* = 1,…, *n*) subject is denoted as *g*_*irKK*_. For instance, when *K* = 1 and *r*_*K*_=*r*_1_, then *g*_*ir*_*K*_*K*_=*g*_*ir*_1_1_ denotes the genotype of the *r1*-th SNV in the gene, where *r1* ranges from 1 to *q1* with *q1* being the number of SNVs in the gene. When *K* = 2 and *r*_*K*_=*r*_2_, then *g*_*ir*_*K*_*K*_=*g*_*ir*_2_2_ denotes the copy number status of the *r2*-th segment in this gene, where *r2* ranges from 1 to *q2* with *q2* being the number of CNV segments in the gene. Quantities *q1* and *q2* may differ from gene to gene. For SNVs, *g*_*ir11*_ is coded as 0, 1, or 2 based on the additive model assumption, whereas *g*_*ir22*_ is coded as 1 for copy number gain or loss, and 0 if unchanged. Then, at the gene-level, the marker data can be displayed as the matrix **G**.

$$\text{G}={\left({\text{G}}_{i\,r_{K}\,K}\right)}_{n\times \left(q_{1}+q_{2}\right)}$$

(1) $$={\left[\begin{array}{ccc} \begin{array}{ccc} \begin{array}{cc} {\text{g}}_{111}\,{\text{g}}_{121} & \\ {\text{g}}_{211}\,{\text{g}}_{221} & \end{array}\,\cdots \,\begin{array}{c} {\text{g}}_{1\,q_{1}\,1}\\ {\text{g}}_{2\,q_{1}\,1} \end{array} & & \\ \vdots \,\ddots \,\vdots & & \\ \begin{array}{cc} {\text{g}}_{n\,11}\,{\text{g}}_{\mathrm{n21}} & \end{array}\,\cdots \,{\text{g}}_{n\,q_{1}\,1} & & \end{array}\,\begin{array}{c} \begin{array}{c} |\\ | \end{array}\\ \begin{array}{c} |\\ | \end{array} \end{array}\,\begin{array}{ccc} \begin{array}{cc} {\text{g}}_{112}\,{\text{g}}_{122} & \\ {\text{g}}_{212}\,{\text{g}}_{222} & \end{array}\,\cdots \,\begin{array}{c} {\text{g}}_{1\,q_{2}\,2}\\ {\text{g}}_{2\,q_{2}\,2} \end{array} & & \\ \vdots \,\ddots \,\vdots & & \\ \begin{array}{cc} {\text{g}}_{n\,12}\,{\text{g}}_{\mathrm{n22}} & \end{array}\,\cdots \,{\text{g}}_{n\,q_{2}\,2} & & \end{array} & & \end{array}\right]}_{n\times \left(q_{1}+q_{2}\right)}$$

where *i* = 1,2,…,*n*,*r*_*k*_ = 1,2,…,*q*_*k*_and *K* = 1,2.

**FIGURE 1:**
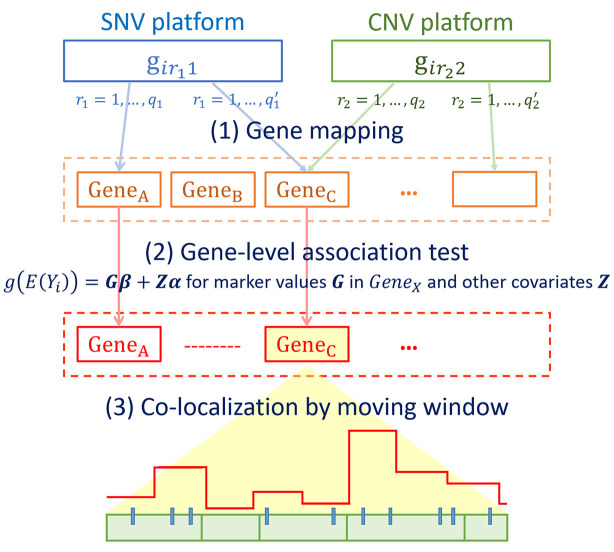
Flowchart of the integrative co-localization (INCO) analysis.

Figure 1 outlines the procedures of INCO. The first step is to map SNVs and CNV segments to the corresponding gene or regulatory regions in order to prepare the matrix **G** for each gene [step (1) in Figure 1]. In step (2), this matrix is included in a gene-level association test to filter for potential target genes. To test for both common and rare variants simultaneously, the SKAT-O test (Lee S. et al., 2012; Ionita-Laza et al., 2013) is utilized. This test follows a generalized linear model approach with the link function *g*(*E*[*Y*_*i*_])=**Gi**β + **Zi**α, where *Yi* is the disease phenotype of the *i*-th subject; αandβ are parameters to be estimated; and **Zi** contains other non-genetic covariates. Note that this gene-level test is a fundamental and crucial step of INCO. The use of the matrix >**Gi** can include simultaneously both types of markers, each coded as a categorical variable, and the SKAT-O test is used with the data matrix evaluated at the gene-level. Genes passing the association test are considered as candidate targets, and are retrieved for further examinations at a finer scale. In step (3), these target genes are investigated with a fine-mapping moving window approach to co-localize the potential causal regions. In each window, the number of genetic components is much smaller and the association test is performed to evaluate the concurrent association in the region. This is the final step of INCO which is carried out at the region-level where the corresponding matrix **Gi** is now evaluated at the region-level, and this completes the integrative co-localization association analysis. Since both types of markers in the **G** matrix are treated equally, and this genetic region is to be tested with the generalized linear model in INCO, we consider it a test of the collective effect from both types of markers. This differs from the parallel analysis in which each omics platform is analyzed separately (Wu et al., 2019).

### Numerical Simulation Experiments

In the following simulation studies, the performance of INCO is evaluated based on genetic components generated from TWB to preserve the relationship between SNVs and CNVs. Specifically, twenty-one genes were first randomly selected and the SNV components and CNV status as well as the segments were retrieved. Table 1 lists each of these selected genes along with its chromosome number, number of SNVs (*q1*), and number of CNV segments (*q2*). Some of the genetic regions were then selected as exerting association with the phenotype, while the others were not. In Part A, the genetic region has a concurrent effect varying from null to strong, with or without a marginal effect from each of its SNVs and CNV segments. In Part B, this setting was extended to two regions, where each region exerts an association of a different degree. The proposed INCO was compared with traditional integrative analysis methods, which identified signals based on the single-platform analysis followed by the naïve union or intersection of the results.

**TABLE 1:** The twenty-one genes considered in the simulation studies.

No.	Chromosome	Gene	*q*_1_	*q*_2_
1	1	*GNB1*	15	10
2	5	*LOC102467217*	7	1
3	4	*PDE5A*	11	1
4	11	*C11orf48*	2	2
5	1	*CTTNBP2NL*	5	2
6	9	*PTCH1*	12	3
7	9	*SLC25A25*	4	3
8	17	*CCL15-CCL14*	9	4
9	6	*CD164*	7	4
10	11	*IGSF22*	10	5
11	3	*CRBN*	9	5
12	12	*VAMP1*	5	6
13	19	*CLEC17A*	7	6
14	5	*DMGDH*	21	7
15	6	*LPA*	24	7
16	15	*CD276*	7	8
17	12	*MED13L*	19	8
18	3	*KBTBD12*	22	9
19	6	*HLA-DQB2*	19	9
20	5	*GM2A*	12	10
21	13	*LRRC63*	20	10

The information contains the chromosome, gene id, number of SNVs ((*q*_1_)), and number of CNV segments ((*q*_2_)). Some variants in the first gene, *GNB1*, are taken as the causal variants in all simulation settings except A1.

#### Simulation Settings

In Part A, the causal region was comprised of one risk SNV (effect size β_1_), one risk CNV segment (with effect size β_2_), and one concurrent effect (expressed as an interaction) of these two variables (effect size was set at β_3_). Individuals were randomly sampled with replacement from TWB, and the corresponding dichotomous disease status was generated based on a logistic regression model,

$$Y_{i}\,\sim \,B\,e\,r\,n\,o\,u\,l\,l\,i\,\left(p_{i}\right),$$

$$\mathrm{logit}\,\left(p_{i}\right)=\beta_{0}+\beta_{1}\,{\text{SNV}}_{1\,i}+\beta_{2}\,{\text{CN}}_{1\,i}+\beta_{3}\,{\text{SNV}}_{1\,i}\times {\text{CN}}_{1\,i}.$$

For the *i*th individual, *Yi* represents the dichotomous disease status and *pi* is the probability of having the disease. The variable SNV_1i_ denotes the genotype of the selected risk SNV with the coding 1 if this subject carries the minor allele and 0 otherwise. The CNV variable CN_1i_ contains the information from the risk segment; it is coded 1 if there is a copy number change and 0 if not. The intercept β_0_ is the disease prevalence, which was set at 0.01 for all settings. The values of (β_1_,β_2_,β_3_) are listed in Table 2 for different settings in Part A. Setting A1 contains the null effect for all variants, as a way to evaluate the false positive rate (FPR) of INCO and competing methods. Settings A2-A4 contain weak marginal effects (main effects of SNV and CNV, respectively) and different levels of interactions (β_3_ ranging from 0.01 to 2) between SNV and CNV. Setting A5 represents the case when both marginal and concurrent effects are moderate or strong. Settings A6-A9 contain the null interaction effect and different levels of main effects. These four settings accompany A1 as different types of the null concurrent effect.

**TABLE 2:** The list of settings and parameter values in simulation Part A, where W stands for weak, M moderate, S strong, and N no effect.

Setting	SNV & CNV effect	Interaction effect	β_1_	β_2_	β_3_
A1	N-N	N	0	0	0
A2	W-W	W	0.01	0.01	0.01
A3	W-W	S	0.01	0.01	1
A4	W-W	S	0.01	0.01	2
A5	M-M	S	0.5	0.5	1
A6	M-M	N	0.1	0.1	0
A7	M-M	N	0.5	0.5	0
A8	S-S	N	1	1	0
A9	S-S	N	2	2	0

The settings for the two causal genetic regions in Part B are similar, but the effect size in each region can be different or even opposite. These two regions can both exhibit null to strong effects, simultaneously or separately. The disease status is generated by the model,

$$Y_{i}\,\sim \,B\,e\,r\,n\,o\,u\,l\,l\,i\,\left(p_{i}\right),$$

$$\text{logit}\,\left(p_{i}\right)=\beta_{0}+\left\{\beta_{1}\,{\text{SNV}}_{1\,i}+\beta_{2}\,{\text{CN}}_{1\,i}+\beta_{3}\,{\text{SNV}}_{1\,i}\times {\text{CN}}_{1\,i}\right\}$$

$$+\left\{\gamma_{1}\,{\text{SNV}}_{2\,i}+\gamma_{2}\,{\text{CN}}_{2\,i}+\gamma_{3}\,{\text{SNV}}_{2\,i}\times {\text{CN}}_{2\,i}\right\}.$$

A representative list of settings used for the values of (β_1_,β_2_,β_3_,γ_1_,γ_2_,γ_3_) is displayed in Table 3, while the remainder of the settings tested is in Supplementary Table 1. For example, in settings B9-B11 the two causal regions exert opposite effects, and in settings B12-B14 the effect sizes of CNV and SNV are different. The sample size was set at 50, 100, or 200 for the disease and healthy group, and the number of replications was 1,000 for each setting in both Part A and B.

**TABLE 3:** The list of selected settings and parameter values in simulation Part B, where W is for weak, M for moderate, S for strong, and N for no effect.

	Region 1		Region 2		(β_*1*_,β_*2*_,β_*3*_,γ_*1*_,γ_*2*_,γ_*3*_)
	SNV & CNV	Interaction	SNV & CNV	Interaction	
B1	W-W	W	W-W	W	(0.01, 0.01, 0.01, 0.01, 0.01, 0.01)
B2	W-W	M	W-W	M	(0.01, 0.01, 0.5, 0.01, 0.01, 0.5)
B3	W-W	S	W-W	S	(0.01, 0.01, 1, 0.01, 0.01, 1)
B5	M-M	W	M-M	W	(0.5, 0.5, 0.01, 0.5, 0.5, 0.01)
B6	S-S	W	S-S	W	(1, 1, 0.01, 1, 1, 0.01)
B8	S-S	S	S-S	S	(1, 1, 1, 1, 1, 1)
B9	W-W	M	W-W	M	(0.01, 0.01, 0.5, −0.01, −0.01, −0.5)
B10	W-W	S	W-W	S	(0.01, 0.01, 1, −0.01, −0.01, −1)
B12	M-N	W	N-M	W	(0.5, 0, 0.01, 0, 0.5, 0.01)
B13	S-N	W	N-S	W	(1, 0, 0.01, 0, 1, 0.01)

#### Traditional Integrative Analysis

The proposed INCO algorithm was compared with two naïve integrative approaches, the intersection and the union. Briefly, these two approaches start with obtaining significant genes based on data from each platform separately, and then the significant genes across different platforms are combined with the union set operation or are filtered with the intersection operation. It is called *union* if a gene is selected because any one of its component SNVs or CNV segments is significant. In contrast, it is called *intersection* if both a SNV and CNV segment in this gene are significant. This is stage 1 of the traditional analyses. In stage 2, these selected significant genes then undergo a moving window analysis to detect if a finer genetic region is associated with the response. In this stage, any window that contains a significant SNV or a significant CNV segment is considered a significant window. Since either SNV or CNV segment can determine the significance of the window, it is always a union operation in stage 2. We therefore call these traditional integrative methods the *traditional union-union* (TUU) analysis and the *traditional intersection-union* (TIU) analysis. Both are classified as parallel integrative analyses.

The intersection approach requires that the selected gene is concurrently identified in multiple platforms, whereas the union approach requires that the gene is identified in at least one platform. Therefore, the traditional union approach TUU tends to be more liberal and may identify more false positives and fewer true negatives. On the other hand, the traditional intersection approach TIU is more stringent and may produce a higher true negative rate.

## Results

### Evaluation of False Positive Rate

To evaluate and compare if the three competing analyses can control type I error, the results under setting A1 are examined. Since the effect sizes under setting A1 are set to zero, no gene or region is associated with the response variable. Therefore, any identified significant findings are false positives. The false positive rates (FPRs) evaluated at the gene- and region-level of INCO, TIU, and TUU under sample sizes of 50, 100, and 200, respectively, are listed in Table 4.

**TABLE 4:** The false positive rates calculated at the gene-level and at the region-level under the simulation setting A1 of null effects.

	Sample size	INCO	Traditional intersection-union, TIU	Traditional union-union, TUU
Gene-level	200	0.047	0.011	0.384
	100	0.059	0.010	0.360
	50	0.085	0.002	0.306
Region-level	200	0.038	0.010	0.034
	100	0.053	0.010	0.031
	50	0.079	0.002	0.004

The proposed INCO maintained the nominal rate when the sample size was 100 or 200, at both the gene- and region-level. Even when the sample size was as small as 50, the FPR of INCO was less than 10%. The other two algorithms, however, were either too conservative or failed to control the false positives. The conservative traditional intersection-union TIU achieved the lowest rate of close to or less than 1% at both the gene- and region-level, while the liberal traditional union-union had high FPRs of around 30–40% at the gene-level but low FPRs of around 3% at the region-level.

### Evaluation of True Positive Rate

**FIGURE 2:**
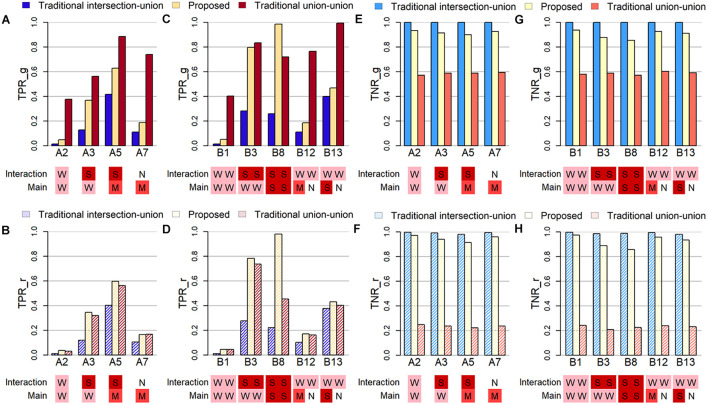
Plots of true positive rates at the gene-level (TPR_g) or region-level (TPR_r) and true negative rates at the gene-level (TNR_g) or region-level (TPR_r) based on selected simulation studies. The letters in colored shades below each figure indicate the degree of effect size of the main and interaction effect in both SNV and CNV. The abbreviations of N, W, M, and S represent Null, Weak, Moderate and Strong, respectively. **(A)** TPR_g in simulation Part A. **(B)** TPR_r in simulation Part A. **(C)** TPR_g in Part B. **(D)** TPR_r in Part B. **(E)** TNR_g in Part A. **(F)** TNR_r in Part A. **(G)** TNR_g in Part B. **(H)** THR_r in Part B.

To examine how well the algorithm can detect the true causal variant, the first criterion is the true positive rate, which is either calculated at the gene-level (TPR_g) or at the causal region-level (TPR_r). The former TPR_g is defined as the average proportion of causal genes that was successfully identified in the simulation studies. The latter TPR_r is defined as the average proportion of causal regions/windows that was identified among all windows containing at least one causal variant. Note that the causal variant can be the SNV and/or the CNV segment, and these two may be located in the same or different regions. Figures 2A–D display the true positive rates (TPRs) under several selected settings in Part A and B, where the sample size is set at 200. Below the X-axis in each subfigure in Figure 2, letters in different colored squares highlight the strength of the corresponding association: W for weak, M for moderate, S for strong, and N for no effect.

When the evaluation is conducted at the gene-level (Figure 2A) and when only one region is causal (Simulation Part A), the traditional union-union, TUU, method outperforms, whether the association is from the marginal SNV or CNV marker and/or from the concurrent effect. However, when the evaluation of TPR is at the region level (Figure 2B), the proposed INCO algorithm becomes the best. This pattern does not change in Simulation Part B when two causal regions are involved, as observed in Figure 2C for TPR_g and Figure 2D for TPR_r. In other words, when the interest lies in detecting finer causal regions, INCO has the best performance through the co-localization technique. Furthermore, it is worth noting that, the TPR of INCO does not change dramatically between TPR_g and TPR_r; its performance is fairly stable. In contrast, TUU and TIU can perform quite differently on the two tasks and are less satisfactory. TIU remains the worse in these comparisons because the intersection criterion is too conservative.

### Evaluation of True Negative Rate

The third criterion used to evaluate the performance is the true negative rate (TNR), which is also calculated at either the gene-level (TNR_g) or at the region-level (TNR_r). The former is defined as the average proportion of non-causal genes (noise) that are not identified as significant over the 1,000 replications, and the latter is defined as the average proportion of non-causal windows that are not detected as signals. Figures 2E–H demonstrate these TNRs. Both INCO and traditional intersection-union performed well, with comparable results, whether the concurrent and marginal effects were weak or strong, and whether this was evaluated at the gene-level (Figures 2E,G) or at the finer region-level (Figures 2F,H). The liberal traditional union-union, however, failed to provide satisfactory results, especially when evaluated at the region-level.

### Application to Taiwan Biobank Data

The Taiwan Biobank project started in 2012 aiming to collect Taiwan population data for large-scale studies of chronic and local diseases. It was designed to recruit 200,000 subjects and is still an ongoing project. Data collected include questionnaires of demographic information, physical examinations, blood and urine tests, and biological samples. Here we consider two biochemical measurements, low-density lipoprotein cholesterol (LDL-C) level, as a continuous phenotype, and triglyceride (TG) level, coded as “optimal” or “high” with a cutoff of 200 mg/dL based on the WHO guidelines (World Health Organization, 2006), as a dichotomous phenotype.

**FIGURE 3:**
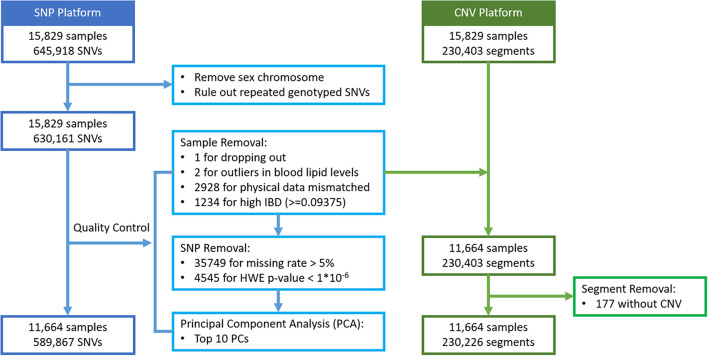
Flowchart of genomic data management for Taiwan Biobank Data.

For 15,829 subjects with genotype data, their SNVs and CNV segments were mapped to a total of 22,427 autosomal genes, based on the hg19 gene list. Based on high identity-by-descent (IBD) values, mismatched physical data, and having unrealistically high blood lipid levels, a total of 4,165 subjects were removed first. The value 0.09375 was set as the threshold of IBD, which is half of the expected IBD value between third and fourth degree relatives (Anderson et al., 2010). In addition, 40,294 SNVs failed to pass the quality control (QC) procedures including missing rate >5% and HWE test *p*-value <1 × 10^–6^. The Principal Component Analysis (PCA) was then carried out on the remaining 589,867 SNVs and the top 10 principal components were extracted to account for possible population stratification. Next, each SNV genotype was coded as 0, 1, or 2 representing the number of minor alleles. All the QC procedures and SNV data recoding were performed with PLINK 1.9 (Purcell et al., 2007; Chang et al., 2015). For CNV segment detection, the following steps were considered: all the raw CEL files were initially examined for quality control with Affymetrix Power Tool (APT) version 1.18.0 and the output summary file was imported to the Partek Genomics Suite 6.6 software for CNV calling. After detection of the individual CNV regions, a segmentation approach was applied, and each segment was recoded as 1 to indicate a change in CNV or 0 to indicate no-change. Among these genes, 72% of them contained both types of markers. Details of the data management are described in Figure 3.

**FIGURE 4:**
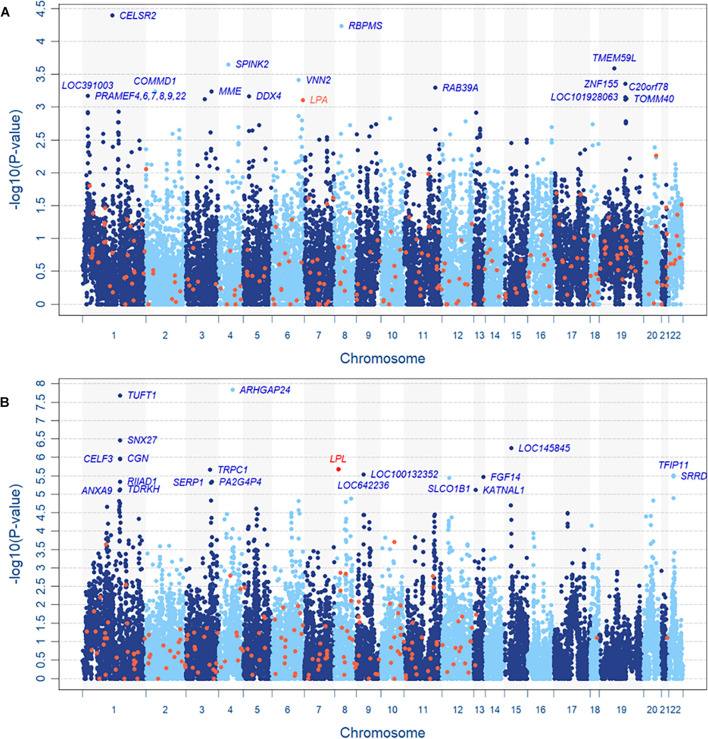
Manhattan plots of TWB, **(A)** continuous LDL-C level and **(B)** dichotomized TG level. In panel **(A)**, *LOC391003*, *PRAMEF4*, *PRAMEF6*, *PRAMEF7*, *PRAMEF8*, *PRAMEF9* and *PRAMEF22* share the same copy number segment which contains no SNV.

Based on the resulting 589,867 SNPs and 230,226 CNV segments in 22,427 genes of 11,664 subjects, the INCO algorithm was performed with the adjustment of covariates including sex, age, body mass index (BMI) and 10 principal components (PCs) for population stratification. Figures 4A,B are the Manhattan plots of step 2 (the gene-level) of INCO for the continuous LDL-C and the dichotomized TG phenotypes, respectively. First, we compare the gene-level results with the associated genes reported earlier. We searched the Online Mendelian Inheritance in Man (OMIM) database (Amberger et al., 2009) and obtained 236 and 185 genes for LDL-C and TG, respectively. These genes are colored in orange in Figures 4A,B. The overlap of the top twenty genes between the set from OMIM and INCO is limited but not inconsistent. For instance, the *lipoprotein* (*A*) (*LPA*) in Figure 4A is identified in OMIM and ranks 23rd by INCO. The *lipoprotein lipase* (*LPL*) gene in Figure 4B is identified by INCO and reported in OMIM. Both *LPA* and *LPL* could be common susceptible genes across populations.

**FIGURE 5:**
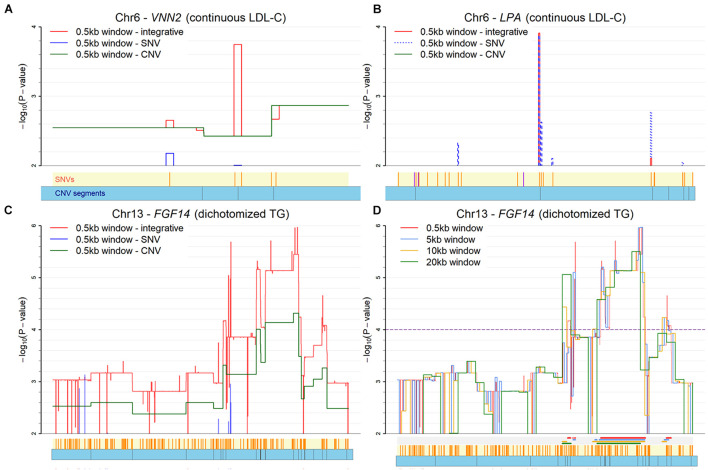
Plot of co-localization analysis. **(A)** The *VNN2* gene for LDL-C. **(B)** The *LPA* gene for LDL-C. **(C)** The *FGF14* gene for TG with three platforms (SNV-only, CNV-only, and both). **(D)** The *FGF14* gene for TG with four window sizes. Below each *X*-axis, the orange ticks inside the yellow region indicate positions of SNVs and the blue bars below it represent the CNV segments. Extra color lines below the *X*-axis in panel **(D)** denote the identified association regions (*p* < 0.0001) under different window sizes.

For the fine-mapping with moving windows, the results for three genes with a window size of 0.5kb are illustrated in Figure 5. The *VNN2* gene for LDL-C is illustrated in Figure 5A. This *VNN2* gene is close to the known *LPA*. In the figure, three skylines for the negative log p-value are used to represent the moving window analysis based on SNV-only, CNV-only, and INCO, respectively. With INCO, the skyline for co-localization peaks around rs1883613 and rs35939522, implying a strong collective association. Note that this region would not be identified with the single-platform analysis, let alone with TUU or TIU. Further investigation of this region demonstrates that samples carrying mutations both from SNV and CNV simultaneously have lower LDL-C values (Supplementary Figure 1B). Similar patterns can be observed in other genes identified by INCO, such as the *TOMM40*, *ZNF155* and *DDX4* genes. The LDL-C values of subjects with or without the mutations are displayed in Supplementary Figures 1A,C,D.

As for the dichotomized status of TG, the odds ratios of the identified regions are calculated to illustrate if individuals carrying mutations in this region tend to have higher risk of higher TG levels. In the calculation, subjects carrying no mutations of an SNV or a CNV in the region are defined as the reference group. The co-localized regions in *TUFT1*, *TFIP11*, *SNX27* and *TDRKH* genes are displayed with the odds ratio plots in Supplementary Figure 2, where subjects with mutations in both an SNV and a CNV segment do show a larger odds ratio than those without mutation and those with just one type of mutation.

## Discussion

In this study, we propose the INCO algorithm to integrate SNV and genomic copy number data in one statistical model for co-localization and identification of associated markers. The novelty targets incorporate data from multi-platforms to account for concurrent effects and to guard against sparsity due to rare variants. The simulation studies suggest that INCO provides a powerful tool for detecting a collective effect of mutants across platforms, as compared with single platform analyses and traditional intersection and union methods, while controlling the false discovery rate at a nominal level. The analyses of real data applications identified associated genes and a region potentially related to phenotypes of interest. The region spans 500 base pairs and can be considered a candidate region for further studies.

For the real application to the Taiwan Biobank database, dietary habits and other environmental factors may vary across populations, which can affect the lipid-related phenotypes more than the genetic contribution to the phenotype. In addition, the Taiwan Biobank project recruited healthy controls without a history of cancer. This could explain why genes identified here do not overlap much with genes reported in earlier studies. Nevertheless, the collective association of the SNV and CNV identified in *VNN2*, as shown in Supplementary Figure 1A, could be a novel finding. *VNN2* is a protein coding gene involved in cell migration and fatty acid metabolism (van Diepen et al., 2014). Studies have shown that the gene expression level of *VNN2* changed in monocytes from hyperlipidemia patients under atorvastatin treatment, which is a prescription medication used to improve cholesterol metabolism (Llaverias et al., 2008). Another study showed that genes, including the *TUFT1* gene, were differentially expressed in liver cells from hyperlipidemia mice under treatment with red raspberry extract. The expression of those genes regulated by the treatment may inhibit liver cholesterol synthesis and accelerate the conversion from triglyceride to fatty acid, which further ameliorates lipid metabolism disorder (Tu et al., 2019). The list of identified genes and regions can be candidates for future investigation of the mechanism.

A limitation of the current TWB application study, however, is that we focus on demonstration of the INCO algorithm for integrative analysis and have not conducted studies to validate the influence of the identified associated regions on the LDL-C and triglyceride level. In addition to advancing this algorithm in the future, collaboration with experts in animal studies would be a means to overcome this limitation.

The applicability of the proposed INCO algorithm can go beyond integrative analysis. For instance, INCO can detect the effects that exist only in one single platform. In Supplementary Figure 3, the INCO procedure is compared with analyses using only SNV or only CNV data, under the scenarios considered in simulation Part A and B. INCO performed as well as the single-platform analysis when the purpose was detection of only main effects. As a demonstration, we performed the co-localization analysis specifically for the *LPA* gene for LDL-C in the Taiwan Biobank data. The skyline displayed in Figure 5B reveals the highest peak around rs73596816, which replicates the findings reported in earlier literature for European and Asian populations (Mack et al., 2017; Han et al., 2019). INCO was able to detect the signals contributed by the single platform alone. Similarly, INCO can be applied directly to finer genetic regions with the moving window approach. That is, if the computational cost is not a concern, the proposed two-stage INCO can be replaced with simply the second-stage of INCO. In Supplementary Figure 4, we compare the one-stage and two-stage co-localization strategies. Both performed similarly under different scenarios and identified the same region. Therefore, two-stage INCO can be used if filtering is required to save computational cost, or its second stage can be applied directly when cost is not a concern.

There are issues in the implementation of INCO that require attention. First, the size of the moving window remains subjective. However, our experience indicates that the identified region is robust to the choice of the size. The influence of the size is demonstrated in the association study of the *FGF14* gene with TG in Figures 5C,D. Four different window sizes (0.5, 5, 10, and 20kb) were considered in this demonstration. It is clear that the patterns of the skylines are similar, implying the robustness of INCO’s findings to window size. Second, INCO relies on a generalized linear model analysis which is parametric. To evaluate if the distribution of the phenotype affects the results of INCO, we have considered three transformations of the original LDL-C and TG measurements as the response variable in the analysis, including continuous rather than binary TG, dichotomized LDL-C with a threshold of 130 mg/dL, and normal versus extremely high LDL-C (≥160 mg/dL). When comparing the findings based on continuous and dichotomized TG, the results are fairly consistent, with up to 90% of the identified genes common to both. As for LDL-C, the degree of overlap is less substantial, but more than 50% of the identified genes are in common. Although INCO performs similarly with these different distributions, a more careful investigation would be required to determine how robust INCO can be to various distributional assumptions.

Third, other localization methods may be adopted in this integrative analysis. The current moving window represents an intuitive and convenient choice. Many current fine-mapping methods have been developed in the post-GWAS era. However, methods such as fGWAS and CAVIAR are designed mainly for locus-specific data instead of structural variants (Spain and Barrett, 2015). Besides, Bayesian fine-mapping methods with functional information scores for SNVs from different databases are usually incorporated into analysis, but few scoring systems are universally useful for copy number variations. It is promising to extend INCO to sequencing data which have higher resolution for finer localization to explore novel cancer-driving mutations. Fourth, since one advantage of INCO is to screen, in stage 1, the potential markers to enter the analysis in stage 2. The current design of INCO is not suitable for a joint model with high-dimensional markers tested at the same time. That is, if the total number of markers from different omics platforms is large, then it would be better to replace the SKAT-O test with some variable selection procedures. Wu et al. (2019) gave a comprehensive review of integrative analysis from the perspective of variable selection, and methods discussed there would be good candidates.

Similar to the above issue, INCO does not test for interaction between SNVs and CNV segments because the test applied here is for genetic association of the region. Although the point estimate of each coefficient in the generalized linear model can be derived, a statistical interaction test has to incorporate the consideration of the significance of the main effect, specification of the form of interaction, and degree of the association due to the interaction term. Zhou and colleagues (Zhou et al., 2021) have reviewed methods from this perspective and provided good references therein. Finally, the ideas of INCO can be applied to other omics data. In this research we have demonstrated the INCO algorithm with SNV and CNV data. When other omics platforms are of interest, the same idea can be applied. The only difference would be the statistical tests adopted at the gene-level and at the region-level investigation. For instance, if transcriptome (microarray or next-generation sequencing) or DNA methylation profiling is considered, then association tests for continuous data, such as the N-statistics (Glazko and Emmert-Streib, 2009), would be more appropriate than the SKATO test; or if RNA-seq data is considered, then other association tests for count data, such as the generalized linear model (McCarthy et al., 2012), would be a more appropriate choice.

## Data Availability Statement

The data analyzed in this study is subject to the following licenses/restrictions: One can apply to access Taiwan Biobank Data. Requests to access these datasets should be directed to biobank@gate.sinica.edu.tw.

## Ethics Statement

The studies involving human participants were reviewed and approved by Institutional Review Board, Taichung Veterans General Hospital, Taichung, Taiwan. The patients/participants provided their written informed consent to participate in this study.

## Author Contributions

Q-YY, T-PL, and CH conceived the study design and draft the manuscript. Q-YY conducted the simulation studies and real data analysis. T-PL, T-HH, C-HL, and CH supervised the data analysis and simulation designs. C-YW and J-YT conducted the CNV management and supervised the integrative analysis. All authors approved the manuscript.

## Conflict of Interest

The authors declare that the research was conducted in the absence of any commercial or financial relationships that could be construed as a potential conflict of interest.

## Publisher’s Note

All claims expressed in this article are solely those of the authors and do not necessarily represent those of their affiliated organizations, or those of the publisher, the editors and the reviewers. Any product that may be evaluated in this article, or claim that may be made by its manufacturer, is not guaranteed or endorsed by the publisher.
