# Supplementary material for: An Integrative Co-localization (INCO) Analysis for SNV and CNV Genomic Features With an Application to Taiwan Biobank Data
Source: Front Genet. 2021 Sep 8;12:709555. doi: 10.3389/fgene.2021.709555 (PMC8456116; doi:10.3389/fgene.2021.709555)
Supplement: Supplementary file 1 [file Presentation_1.pdf]

Supplementary Material

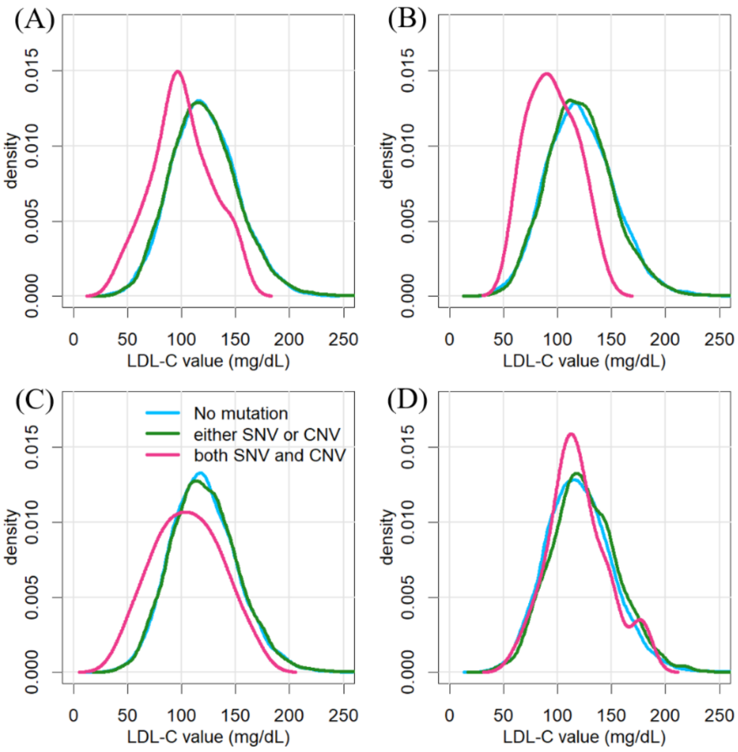

Supplementary Figure 1. Density plots of the association regions. (A) *DDX4* gene (window of chr5:55,094,344-55,094,843) with rs3761778; (B) *VNN2* gene (chr6:133,077,008-133,077,507) with rs1883613 and rs35939522; (C) chr19 *ZNF155* gene (chr19:44,501,321-44,501,820) with rs446016; (D) chr19 *TOMM40* gene (chr19:45395476-45395975) with rs2075650. The genomic locations are the co-localized region by moving-window analysis with the window size of 0.5kb.

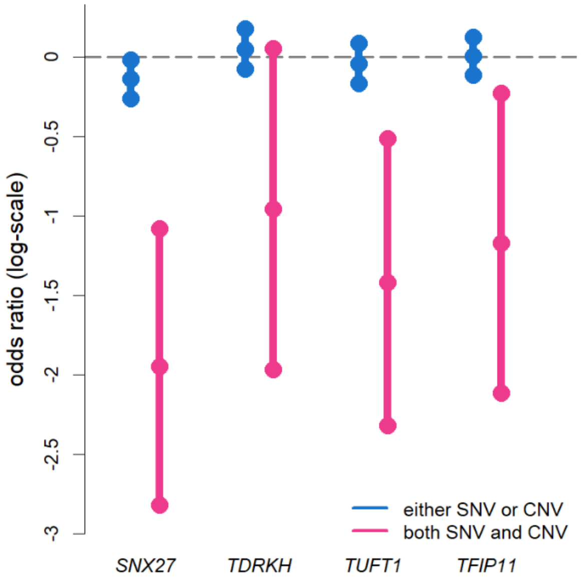

Supplementary Figure 2. Plots of Odds ratios and 95% confidence intervals for the *SNX27* gene (chr1:151,668,161-151,668,660with rs1308137), *TDRKH* gene (chr1:151,751,540-151,752,039 with rs11204886), *TUFT1* gene (chr1:151,520,280-151,520,779 with rs6587597), *TFIP11* gene (chr22:26,899,393-26,899,892 with rs134136). The genomic locations are the moving windows identified by INCO. The window size is 0.5kb.

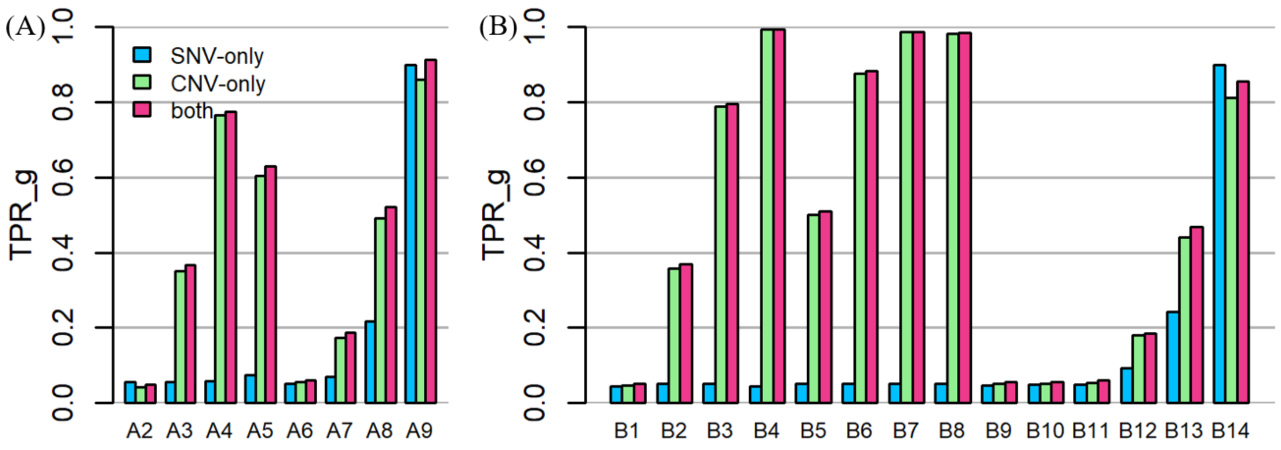

Supplementary Figure 3. Comparison between INCO and two association tests with only one single platform data. The measure of evaluation is the true positive rate at the gene-level (TPR_g).

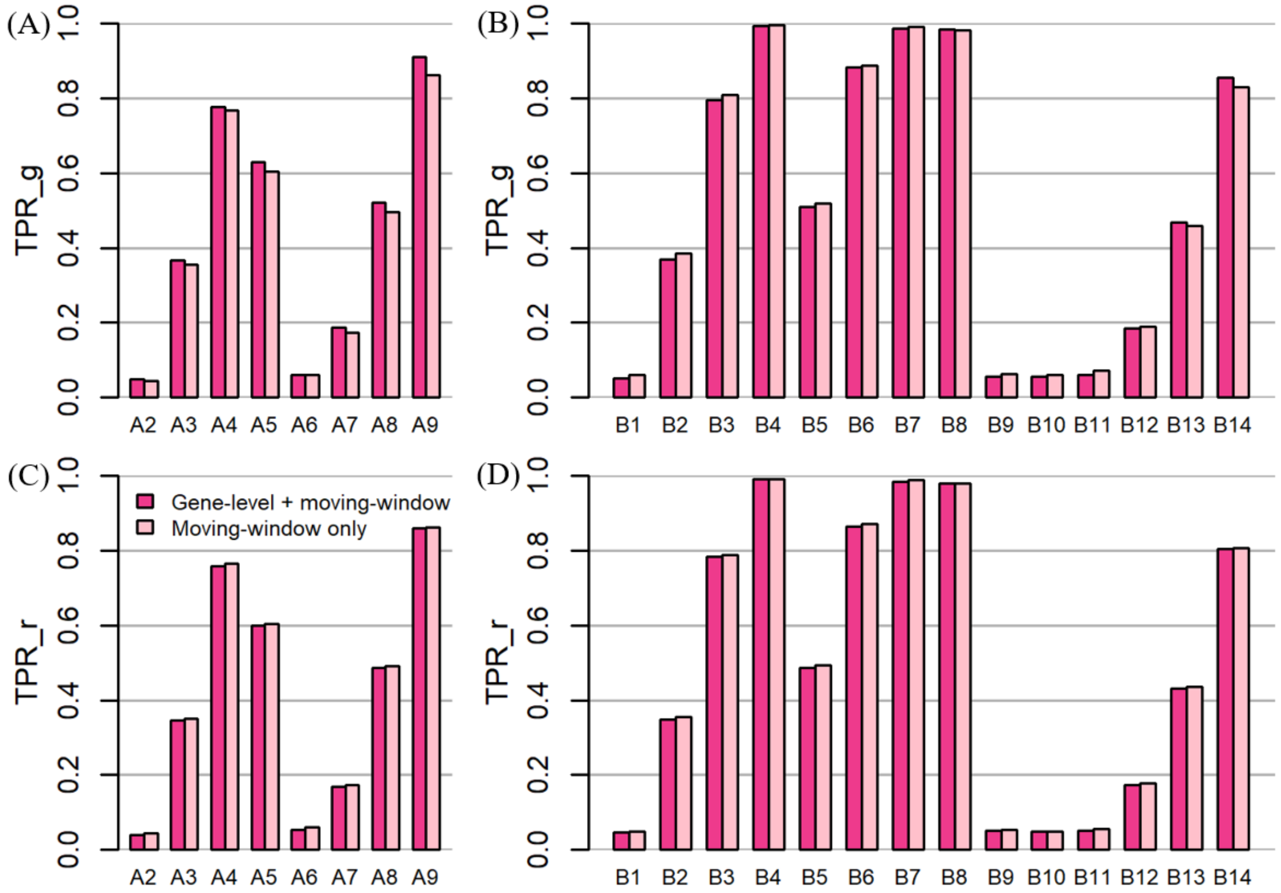

Supplementary Figure 4. Plots of TPR for simulation Parts A & B. The comparisons between one-stage (moving-window only) and two-stage (gene-level and moving-window) analyses with true positive rate at the gene-level (TPR_g) and region-level (TPR_r).

Supplementary Table 1. The complete list of settings and parameter values in simulation Part B, where W for weak, M for moderate, S for strong, and N for null effect.

	Region 1		Region 2		$(\beta_1, \beta_2, \beta_3, \gamma_1, \gamma_2, \gamma_3)$
	SNV & CNV	interaction	SNV & CNV	interaction	
B1	W-W	W	W-W	W	(0.01, 0.01, 0.01, 0.01, 0.01, 0.01)
B2	W-W	M	W-W	M	(0.01, 0.01, 0.5, 0.01, 0.01, 0.5)
B3	W-W	S	W-W	S	(0.01, 0.01, 1, 0.01, 0.01, 1)
B4	W-W	S	W-W	S	(0.01, 0.01, 2, 0.01, 0.01, 2)
B5	M-M	W	M-M	W	(0.5, 0.5, 0.01, 0.5, 0.5, 0.01)
B6	S-S	W	S-S	W	(1, 1, 0.01, 1, 1, 0.01)
B7	S-S	W	S-S	W	(2, 2, 0.01, 2, 2, 0.01)
B8	S-S	S	S-S	S	(1, 1, 1, 1, 1, 1)
B9	W-W	M	W-W	M	(0.01, 0.01, 0.5, -0.01, -0.01, -0.5)
B10	W-W	S	W-W	S	(0.01, 0.01, 1, -0.01, -0.01, -1)
B11	W-W	S	W-W	S	(0.01, 0.01, 2, -0.01, -0.01, -2)
B12	M-N	W	N-M	W	(0.5, 0, 0.01, 0, 0.5, 0.01)
B13	S-N	W	N-S	W	(1, 0, 0.01, 0, 1, 0.01)
B14	S-N	W	N-S	W	(2, 0, 0.01, 0, 2, 0.01)
